# The Therapeutic Benefit of Bacterial Membrane Vesicles

**DOI:** 10.3390/ijms18061287

**Published:** 2017-06-16

**Authors:** Natalie J. Bitto, Maria Kaparakis-Liaskos

**Affiliations:** 1 Department of Physiology, Anatomy and Microbiology, La Trobe University, Bundoora, Melbourne, Victoria 3086, Australia; n.bitto@latrobe.edu.au; 2 Centre for Innate Immunity and Infectious Diseases, Hudson Institute of Medical Research, Monash University, Melbourne, Victoria 3068, Australia

**Keywords:** bacterial membrane vesicles, vaccine design, cancer therapy, recombinant bacterial membrane vesicles

## Abstract

The therapeutic potential of extracellular vesicles from eukaryotes has gained strong interest in recent years. However, research into the therapeutic application of their bacterial counterparts, known as bacterial membrane vesicles, is only just beginning to be appreciated. Membrane vesicles (MVs) from both Gram-positive and Gram-negative bacteria offer significant advantages in therapeutic development, including large-scale, cost effective production and ease of molecular manipulation to display foreign antigens. The nanoparticle size of MVs enables their dissemination through numerous tissue types, and their natural immunogenicity and self-adjuvanting capability can be harnessed to induce both cell-mediated and humoral immunity in vaccine design. Moreover, the ability to target MVs to specific tissues through the display of surface receptors raises their potential use as targeted MV-based anti-cancer therapy. This review discusses recent advances in MV research with particular emphasis on exciting new possibilities for the application of MVs in therapeutic design.

## 1. Introduction

The ubiquitous production of extracellular vesicles by bacteria is now widely accepted as a novel bacterial secretion system [1]. These membrane-bound nanostructures, known as membrane vesicles (MVs), are non-replicative and are produced during all stages of growth in vivo and in vitro by both Gram-negative and Gram-positive bacteria alike [2]. It is suggested that MVs have played important roles since the beginnings of cellular evolution, as evidence suggests that MVs released by primitive intracellular bacteria led to the formation of cellular organelles such as the Golgi body and endoplasmic reticulum [3]. Bacteria utilize MVs as delivery vehicles that facilitate a multitude of biological functions, including communication and competition [4,5], biofilm formation [6,7], survival under environmental stress [8], and pathogenesis [9,10,11]. Exploiting this natural delivery system of bacterial MVs for therapeutic benefit has proven to be safe and effective as a commercial vaccine [12,13], and is highly promising for the development of novel therapies.

## 2. Structure and Composition of MVs

**Figure 1:**
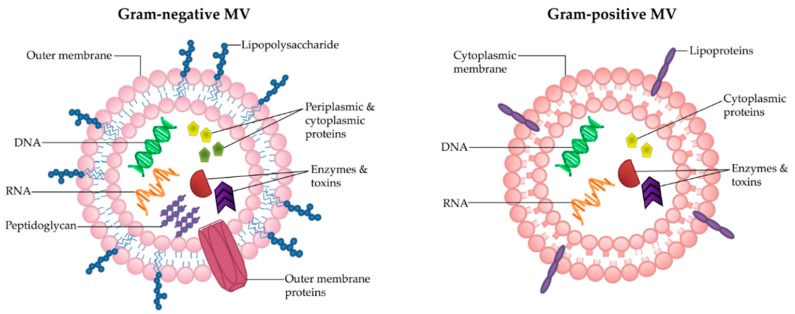
Architecture and composition of Gram-negative and Gram-positive MVs. While MVs are heterogeneous in size and contents, typically Gram-negative MVs are encased in the outer membrane which is embedded with LPS and outer-membrane proteins, and carry periplasmic contents including peptidoglycan, enzymes and toxins, as well as cytoplasmic proteins and nucleic acids. Gram-positive MVs are comprised of the cytoplasmic membrane and have been reported to contain lipoprotein, cytoplasmic proteins, enzymes, toxins and nucleic acids.

MVs are natural carriers of small molecules, including proteins, enzymes and nucleic acids [14]. They are heterogeneous in size and contents, ranging from 20–300 nm [1]. Bacterial MVs carry a range of cargo, including lipopolysaccharide (LPS) [15,16], peptidoglycan [11,17,18], membrane, periplasmic and cytoplasmic proteins [19,20,21,22,23,24], toxins [25,26] and nucleic acids [27,28,29,30,31]. There are also reports identifying that bacterial cargo can be selectively included into MVs [32,33,34,35]. Due to the differences in parent cell architecture, there are subtle variances between the biogenesis and composition of Gram-positive and Gram-negative MVs (reviewed in [2,36] (Figure 1). Gram-negative bacteria possess an outer membrane, which encases the MVs that they shed and thus they are also known as “outer-membrane vesicles” (OMVs). OMVs are produced by multiple mechanisms including via blebbing from the outer membrane [37] and as a result of LPS remodeling [38], as reviewed in [14,39]. Recent findings in the literature suggests that OMVs can be produced by spontaneous explosive cell lysis, whereby membrane fragments of the lysed bacteria form closed vesicles that engulf the surrounding cellular components [40]. This latter process may be a method whereby bacterial DNA is incorporated into OMVs [40].

In contrast to Gram-negative bacteria, the membranes of Gram-positive bacteria are surrounded by a thick and rigid cell wall, which for many years was thought to prevent the release of MVs [41]. As a result, Gram-positive MVs have been largely overlooked [2]. However, in 2009 electron microscopy confirmed the release of bilayered MVs from *Staphylococcus aureus* [41]. Subsequently, other studies have shown MV release from a range of Gram-positive bacteria including *Bacillus anthracis* [42], *Listeria monocytogenes* [43], *Clostridium perfringens* [44], *Streptococcus sp.* [35,45], and *Bacillus subtilis* [46]. Analysis of the composition of Gram-positive MVs has shown that, much like Gram-negative MVs, they carry a variety of proteins [35,44], nucleic acids [35,44,45,47] and toxins [42,44]. In fact, both share analogous biological roles in inter-bacterial communication, bacterial survival, and host-pathogen interactions [41,43,48,49]. The mechanism of Gram-positive MV release through the thick cell wall remains elusive, however, the presence of peptidoglycan-degrading enzymes in Gram-positive MVs suggests that the cell wall may be altered for MV release [41].

## 3. Internalization of MVs into Host Cells

MVs have the ability to directly interact with host cells through internalization and release of their contents within the cell, as reviewed in [1] and [14]. MV internalization has been widely reported to be dependent on lipid rafts [10,11,50], and in some cases this requires the engagement of other cellular receptors [51]. For example, entry of MVs from *Helicobacter pylori* [11], *Aggregatibacter actinomycetemcomitans* [50], and *Pseudomonas aeruginosa* [10] into host cells is dependent on lipid raft microdomains in the plasma membrane. Similarly, membrane fusion of enterotoxigenic *Escherichia coli* MVs is lipid raft dependent but also requires interaction between heat-labile enterotoxin on the MVs and its cellular receptor, monosialoganglioside [51]. This is an example of targeted specificity of MVs to cells expressing a particular receptor.

MVs have also been reported to be engulfed by the recipient cell through an active process called endocytosis [16,51,52,53,54]. There are three types of selective endocytic pathways in non-phagocytic cells: clathrin-mediated endocytosis, caveolin-mediated endocytosis, and caveolin-/clathrin-independent endocytosis, reviewed in [55]. Various studies have collectively demonstrated that each of these pathways are utilized for MV entry into non-phagocytic cells; *H. pylori* MVs have been observed to use both clathrin-dependent and clathrin-independent mechanisms [52,53], enterotoxigenic *E. coli* MVs enter through clathrin-mediated endocytosis [51], while *Porphyromonas gingivalis* MVs use a caveolin-/clathrin-independent process [54] to enter host cells. Overall, non-phagocytic MV internalization appears to occur through an array of different mechanisms, which is thought to be bacterial species specific [1].

The ability of MVs to enter eukaryotic host cells and carry their cargo within makes them ideal long-distance delivery vehicles. Bacteria use this feature for intracellular communication, pathogenesis, and regulating host immunity for their benefit; however, it can also be exploited for therapeutic applications to treat or prevent bacterial infections, as discussed below.

## 4. Advantages of MVs in Therapeutic Applications

MVs have a number of qualities that make them advantageous for biopharmaceutical applications. They are cost-effective to produce, temperature-stable, and have shown to be safe when used as a human vaccine [56].

MVs can be readily produced in inexpensive liquid media from large-scale cultures of Gram-negative bacteria, Gram-positive bacteria, or mycobacteria [57,58]. Bioreactors have been successfully used to upscale *Neisseria meningitidis* MV production [59], while the use of hyper-vesiculating mutants has been shown to greatly increase the yield of MVs [60,61]. The MV isolation process, reviewed in [57] is relatively straightforward and inexpensive, involving the removal of bacteria by centrifugation and subsequent filter sterilization through a 0.22 µm filter, followed by ultracentrifugation at 100,000× *g* to pellet the MVs [57]. This MV preparation may contain other bacterial components such as pilli, flagella or cellular debris, therefore to ensure sample purity it is necessary to carry out further purification [57]. Gradient ultracentrifugation separates MVs from non-MV contaminants based on size using a density gradient solution such as OptiPrep [57] or sucrose [11]. Affinity purification enables isolation of MVs that contain a particular protein of interest, such as a histidine tag [62]. Accurate quantification of MVs can be carried out using particle counting systems such as NanoSight [63] or flow cytometry [64], both of which count individual MVs based on size.

The lipid membrane surrounding MVs offers protection of the internal cargo. MVs protect their encased cargo from degradation by nucleases [29,65] and proteases [66,67,68,69]. MVs have been shown to retain enzymatic activity and antigenicity over long-term storage at 4 °C [70,71], as well as short exposure to elevated temperatures. A study has shown that an enzyme, phosphotriesterase (PTE) packaged into *E. coli* MVs retained enzymatic activity over 100-fold greater than free PTE when subject to elevated temperatures (37 °C for 14 days), and multiple freeze-thaw cycles [66]. Another study that evaluated the antigenicity of *N. meningitidis* MVs under different storage conditions showed that MVs could be stored in liquid form at 4 °C for a year with no loss in antigenicity, however storage at 37 °C or higher for three months resulted in compromised antigenicity [71]. These studies highlight the long-term stability of MVs.

MVs have been used safely and effectively as the main component of the licensed meningococcal serogroup B vaccine [13]. Similar OMV-based vaccines produced from *N. meningitidis* are currently licensed in 37 countries worldwide to prevent meningococcal disease [72]. Their efficacy, tolerability and safety has been widely reported [12,73,74,75,76]. In addition, the meningococcal serogroup B vaccine has been proven safe and effective in children as young as two years of age [73]. The success of the meningococcal serogroup B vaccine highlights the potential of MVs as a safe and economically viable vaccine platform and has paved the way for the future development of other MV-based therapeutics.

## 5. MVs Drive Innate and Adaptive Immune Responses

MVs naturally contain a range of highly immunostimulatory ligands known as microbial-associated molecular patterns (MAMPs), such as LPS, peptidoglycan and bacterial DNA [1]. MAMPs are recognized by pathogen recognition receptors (PRRs) found on epithelial cells and immune cells. The activation on PRRs induces an innate immune response, which functions as the host’s first line of defense [77]. This response is characterized by the production of pro-inflammatory cytokines and chemokines, followed by the recruitment of immune cells [77]. The MAMPs carried by MVs, and their ability to activate PRRs expressed on the host cell surface and intracellularly, makes MVs strong drivers of the innate immune response [1]. Moreover, as OMVs serve as vehicles that can carry MAMPs distally throughout the body, they are inherently effective at activating a systemic innate immune response in the host [78,79]. A plethora of studies have also reported the ability of MVs to induce an adaptive immune response, as reviewed in [1]. Below, we focus on select studies that highlight the ability of MVs to induce a long-lasting humoral and cellular immune responses.

MVs have been shown to induce long-lasting humoral immune responses. An in vivo study characterizing the *N. meningitidis* MV vaccine showed it induced long-lasting memory B and T-cells for 120 days in mice [80]. The antibody profile consisted of multiple isotypes including IgA, IgM, IgG1, IgG2a, IgG2b, and IgG3 and serum displayed bactericidal activity [80]. Another study found that *N. meningitidis* MVs derived from an isolate naturally expressing key antigenic membrane proteins were cross-reactive against four *N. meningitidis* strains [81]. Furthermore, responses were long-term of up to 92 days in rabbits and antibodies generated in response to these MVs were bactericidal [81]. Similarly, studies examining the humoral response to *Vibrio cholerae* MVs have demonstrated high-titer IgG, IgM, and IgA antibodies with long-lasting protection for three months in mice [82,83]. Oral and intranasal routes of administration were most effective at producing IgA antibodies required for mucosal immunity against *V. cholerae* MVs [82]. Investigation into the mechanism of action of the antibodies generated as a result of vaccination with *V. cholerae* MVs showed that they were not bactericidal but rather inhibited bacterial motility [84].

Furthermore, the humoral immune response generated in response to Gram-positive MV vaccines has also been investigated, with varying success. Vaccination of mice with *Streptococcus pneumoniae* MVs elicited antibody production that was protective against pneumococcal infection [85], and vaccination of mice with *S. aureus* MV conferred protective cell-mediated and humoral immunity against staphylococcal lung infection [86]. Conversely, vaccination of mice with *C. perfringens* MVs caused high-titer IgG antibody production when administered intraperitoneally, but these antibodies were not protective against challenge with a lethal dose of *C. perfringens* [44].

Since MVs reflect the structure of the bacterial membrane, they therefore can display antigens in a native conformation that is more effective at producing neutralizing antibodies than purified antigen alone [87]. A study in mice comparing the immunogenicity of *N. meningitidis* MVs carrying the antigenic porin protein PorA, compared to purified PorA protein, found that while antibodies were produced in response to both, only the ProA-containing MVs induced bactericidal antibodies [88]. This finding highlights the advantage of MVs in presenting antigens in their native state to induce an effective immune response.

Cell-mediated immunity plays a vital role in clearing the invading pathogen from the host [89], and MVs are highly efficient at inducing the development of cell mediated immunity. Immunization of mice with *E. coli* MVs showed protection from lethal challenge was primarily due to T-cell immunity, particularly Th1 and Th17 cell and cytokine (IFN-γ and IL-17, respectively) responses [90]. Vaccination of mice with *V. cholerae* MVs generated a T-cell response including cytotoxic T-cells, Th1, Th17, and regulatory T-cells [91]. Another study identified the generation of long term memory T-cells from mice vaccinated with *N. meningitidis* MVs [80]. MVs from *S. aureus* were shown to induce a Th1 response in mice that was protective against lethal challenge [86]. Other studies have shown that MVs induce a T-cell response including Th1, Th17, and cytotoxic T-cells [92,93,94,95].

Taken together, these studies highlight the ability of MV-based vaccines to induce a protective cell-mediated and humoral immune response. However, it should be considered that there are bacterial species-related differences in the effectiveness of MV-based vaccines that may affect the type of immune response generated and the ability to confer protection.

## 6. Native MV Vaccines

There is a great demand for new vaccines against bacterial pathogens, particularly due to the rise in antibiotic resistance. MVs from numerous bacteria have been investigated as vaccine candidates due to their immunogenic properties, with MVs from both Gram-positive and Gram-negative bacteria showing promise in early vaccine development studies, as reviewed in [14].

Demand for a more effective *Bordetella pertussis* vaccine is increasing due to the recent rise in the incidence of whooping cough [96]. Recent studies have evaluated novel *B. pertussis* MV vaccines compared to the current approved whole cell *B. pertussis* vaccine, and showed that the MV vaccine raised antibody levels in mice comparable to the whole cell vaccine [23,97,98]. Moreover, the MV vaccine was more effective against a current circulating isolate than the whole-cell vaccine [98].

The rise in nosocomial infections due to the multi-drug resistant pathogen *Acinetobacter baumannii* has prompted investigation into the development of an effective vaccine [99]. Studies have shown that vaccination with *A. baumannii* MVs was effective at increasing the survival of mice in a sepsis model [100,101] and reduced bacterial burden and lung inflammation in a pneumonia model [100]. Mice vaccinated with MVs displayed a stronger antibody response compared to mice vaccinated with killed bacteria or purified outer membrane complexes, without the requirement an adjuvant [100]. This study highlights the potential of a self-adjuvanting MV-based vaccine against *A. baumannii*.

Similarly, increasing antibiotic resistance in cases of Staphylococcal infections has prompted *S. aureus* vaccine studies [86]. Previous attempts at *S. aureus* vaccine design have failed as they induced a strong B-cell response but lacked the T-cell response required to clear the pathogen [102]. However, it is reported that vaccination of mice with *S. aureus* MVs resulted in strong cell-mediated and humoral immune responses, which together were effective at conferring protection against lethal challenge, without the use of adjuvants [86].

These studies highlight the potential of novel MV vaccines due to the broad range of immune responses they induce, which in some cases appears to make them more effective than traditional vaccine alternatives. Furthermore, the self-adjuvanting properties of MVs circumvent the need for additional adjuvants, which can have adverse side effects. As there is a limitation of adjuvants suitable for human use, in particular effective mucosal adjuvants, the self-adjuvanting properties of MVs makes them useful as stand-alone vaccines or in conjunction with other vaccines to boost their immunogenicity.

## 7. MVs as Vaccine Adjuvants

Due to their ability to activate the innate immune system, MVs have also proven to be effective as adjuvants used in conjunction with other vaccines. Conventional adjuvants—such as alum, cholera toxin, and diphtheria toxin—often display adverse side effects including inflammation, toxicity, and poor stimulation of cell-mediated or mucosal immunity [103,104]. The inherent components of MVs activate sensors of the innate immune system, such as the activation of Toll-like receptor 4 (TLR4) [15] and the inflammasome [16] by LPS, and activation of nucleotide-binding oligomerization domain-containing protein 1 (NOD1) by peptidoglycan [11]. Furthermore, as MVs are highly effective at inducing cell-mediated and mucosal immunity [73,82], they are being investigated as adjuvants for live and inactivated vaccines, as well as purified antigens [91,105]. A study investigating the adjuvant potential of *E. coli* MVs compared to cholera toxin to enhance a purified malaria antigen vaccine showed that *E. coli* MVs administered intranasally to mice raised antibody titers and cellular responses against the malarial antigen to a level comparable to cholera toxin, with no significant side effects or weight loss [91]. Similarly, intranasal vaccination of mice with an *E. coli* MV adjuvant used in conjunction with a novel purified influenza antigen generated higher titer mucosal antibodies and cell-mediated responses, compared to mice vaccinated intranasally with the purified influenza antigen alone [105]. Furthermore, no adverse effects were associated with the MV adjuvant [105]. These studies showcase the potential of MVs as mucosal adjuvants to improve antibody titer and provide an effective cell-mediated immune response, without the adverse effects of other adjuvants.

## 8. Bioengineered MVs

Since many bacteria can be genetically modified by simple molecular techniques, there is enormous potential to modify bacteria to produce MVs with specific cargo. Controlling the contents of MVs through bioengineering has significantly increased the potential of MV therapies, as reviewed in [106]. Genetic manipulation of the parent bacteria can refine the functionality of MVs, for instance it allows directed packaging of recombinant epitopes, inclusion of signal molecules for cell-specific targeting or exclusion of undesired and toxic MV components such as LPS [56,76,107]. This relative ease of biomanipulation is leading to the development of unique recombinant MV therapies [70].

While Gram-negative MV vaccines are potentially safe for human administration, this is not without first removing the toxic LPS component [76]. Also known as endotoxin, LPS carried by MVs from Gram-negative bacteria has been shown to be more potent than free LPS and can lead to septic shock [108]. The current meningococcal vaccine achieves LPS detoxification through detergent treatment of the bacteria before production of MVs [76]. However, this can also be done by genetic manipulation to generate a bacterial strain that produces a low toxicity variant of LPS [76]. Studies have shown the efficacy of this technique in MV production for vaccine development from *E. coli* [61,109] and *N. meningitidis* [107]. Furthermore, Gram-positive bacteria that do not carry LPS and have been shown to be well tolerated in vivo [86]. Therefore, it is feasible that MVs from such bacteria could be exploited as a platform for recombinant vaccine production without the need for LPS detoxification.

**Figure 2:**
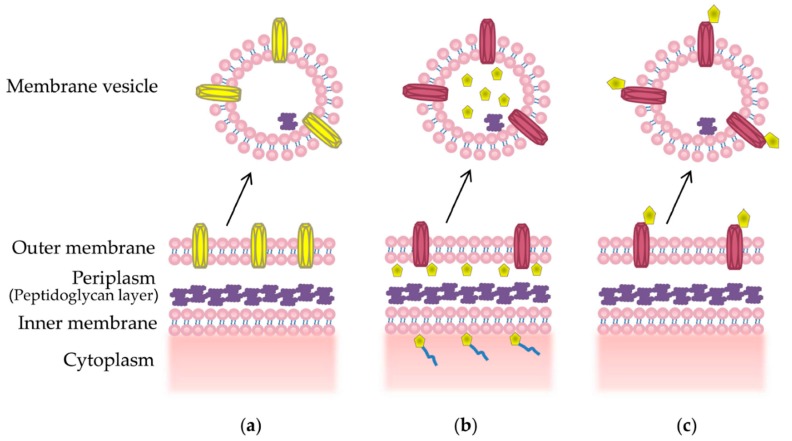
Methods of packaging recombinant proteins into MVs. (**a**) Overexpression of a recombinant membrane protein (yellow) may lead to enrichment on the MV surface; (**b**) A periplasmic signal sequence (blue) can be used to direct the protein of interest (yellow) into the periplasm, whereby the protein of interest may become encapsulated into budding MVs; (**c**) Fusion of a protein of interest (yellow) with a membrane protein can lead to targeted display on the surface of MVs.

Packaging of desired epitopes into MVs can be achieved through a number of methods, reviewed in [76]. Firstly, overexpression of a recombinant outer membrane protein can result in increased packaging into MVs [110] (Figure 2a). This has been successful in displaying a *N. meningitidis* outer membrane protein vaccine candidate, NspA, in its native conformation on the surface MVs from the commensal bacteria *Neisseria flavescens* [110]. Other methods include using a signal sequence that directs the protein of interest to the periplasmic space, where it is encapsulated into budding MV [111] (Figure 2b). This approach was used to encapsulate functional green fluorescence protein (GFP) into *E. coli* MVs using the Tat signal sequence [111]. Surface display of recombinant proteins, which is required for the display of many surface antigens, may be achieved through fusion to a surface anchor protein, such as an abundant outer membrane protein or glycoprotein [70,112] (Figure 2c). This results in localization of the antigen to the MV surface, where it can be efficiently presented to the immune system [113].

Furthermore, careful selection of the anchor protein is required to ensure effective incorporation and display of the recombinant epitope without disrupting MV formation or growth of the parent bacteria [70]. The first study to prove this concept demonstrated that several heterologous proteins could be displayed on the surface of *E. coli* and *Salmonella enterica* MVs by genetically fusing them to the surface protein cytolysin A (ClyA) [114]. Fully functional, recombinant beta lactamase, GFP, and anti-digoxin antibody were fused to the C-terminus of ClyA and secreted into vesicles, highlighting the flexibility of recombinant protein display on the MV surface [114]. ClyA is secreted on the surface of *E. coli* MVs and therefore provides an ideal scaffold to attach recombinant proteins and antigens [114]. A subsequent study showed that MVs expressing recombinant GFP fused to ClyA in *E. coli* induced an antibody response directed to GFP in immunized mice [115]. These seminal advances have paved the way for the development of next generation therapies that may take advantage of the genetic flexibility offered by recombinant MVs.

## 9. Recombinant MV Vaccines

Novel recombinant MV vaccines, though in the early stages of development, have shown great promise. An early example of a recombinant MV vaccine was based on *S. enterica* bacteria engineering to express the *S. pneumoniae* pneumococcal protein, PspA, coupled to a periplasmic signal sequence [116]. The resulting MVs carried the PspA antigen in their lumen, and moreover, conferred protective immunity against *S. pneumoniae* when administered intranasally to mice [116]. Recently, a similar study successfully used the *S. enterica* MV platform to generate protective antibodies against murine pneumococcal infection, this time with an autotransporter system designed to direct a fragment of the *S. pneumoniae* PspA protein onto the MV surface [113]. This approach may be more appropriate for antigens that require surface presentation to retain their native immunogenic conformation, but is limited to small proteins or peptides [113].

Recent studies have developed recombinant MV vaccines through surface expression of epitopes using the *E. coli* ClyA protein [114]. One study has fused the *A. baumannii* outer membrane protein 22 (Omp22) to ClyA in DH5α *E. coli* and successfully produced chimeric *E. coli* MVs [117]. These chimeric MVs were administered intraperitoneally to mice and gave rise to Omp22-specific antibodies that were protective against lethal challenge in a murine sepsis model [117]. The first known example of a MV-based anti-viral vaccine was produced in a study that generated recombinant *E. coli* MVs with ClyA fused to the ectodomain of influenza A matrix protein 2 (M2e), a highly conserved domain on the viral surface [118]. The recombinant M2e MV vaccine induced high IgG titers in mice, without the need for adjuvants, and provided complete protection against lethal PR8 influenza challenge in mice [118]. Furthermore, this M2e MV vaccine provided cross-reactive protection against two influenza strains, H1N1 and H3N2 [61]. Collectively, the early success of these novel “designer” vaccines in animal trials shows great promise for the future of MV vaccine development to target a wide range of diseases.

## 10. MVs in Cancer Therapy

The ability to engineer MVs to target a particular cell type makes them an attractive option for cancer therapy, reviewed in [119]. Current chemotherapy options indiscriminately affect all cells of the body, causing severe and often long-lasting side effects [120]. Furthermore, a high dose of chemotherapy is required to achieve an effect on the tumor due to rapid clearance and poor circulating half-life of the cytotoxic agents [121]. Therefore, novel nanoparticle delivery platforms are being increasingly explored as vehicles to deliver drugs, genes, or imaging agents to target cells [121].

Engineered MVs pose several advantages for cancer therapeutics. The protective membrane may shield the MV contents, such as chemotherapeutic agents, from protease or nuclease degradation, thereby increasing the circulating half-life of the drug [119]. Furthermore, cell-specific targeting may prevent the MV-encapsulated drug from disseminating into the body where it can have unwanted cytotoxic effects, but rather ensure it is released only upon entry into the target tumor [119]. Therefore, MVs with cancer-targeting ligands at their surface and anti-cancer drugs within their lumen could potentially lead to a specific and potent anti-tumor therapy with low toxicity [119].

Two recent publications have shifted MV cancer therapy from a theory to a proven concept. The first study by Gujrati and colleagues [122] successfully engineered a tumor-targeting MV loaded with anti-tumor siRNA. As a cell-specific target, they selected the transmembrane receptor HER2, which is overexpressed in a range of cancers including breast, ovarian, and gastric [122]. Using *E. coli* MVs and the ClyA surface display system, they expressed a small molecule with high binding-efficiency to HER2 (an HER2 “affibody”) on the surface of the MVs, thereby introducing tumor-specific targeting capabilities to the MVs [122]. They then used an electroporation method to load MVs with siRNA directed against mRNA from a key cell cycle regulatory gene implicated in cancer [122,123]. In vitro studies verified the ability of the siRNA-laden recombinant MVs to deliver siRNA and subsequently induce cell death in ovarian and breast cancer cell lines [122]. Moreover, in vivo investigation in mice showed high localization of the recombinant MVs in tumor tissue and a 66% reduction in tumor growth, corresponding to a reduced amount of the target protein in the tumor [122].

The second study used *E. coli* protoplast-derived nanovesicles engineered to express the epidermal growth factor (EGF) receptor on their surface as a novel treatment against cancer [124]. Many epithelial tumors are known to overexpress EGF and the EGF receptor is a known cancer therapy target [125]. Recombinant MVs expressing the EGF receptor were loaded with anti-tumor drugs—doxorubicin or idarubicin—and administered subcutaneously to mice [124]. In vitro verification confirmed that the therapeutic vesicles carrying the anti-tumor drugs targeted lung carcinoma cells and displayed cytotoxicity in a dose-dependent manner [124]. In vivo studies in mice demonstrated that the therapeutic MVs localized most strongly in the tumor tissue and were associated with reduction in tumor growth, with no adverse side effects measured by body weight, temperature, or white blood cell levels [124].

## 11. Challenges and Limitations

While the development of MV therapeutics looks promising, this field is currently in its infancy and some issues need to be addressed before their development as therapeutic agents. Firstly, the heterogeneous nature of MVs makes it difficult to ensure the batch to batch consistency required for human application and this needs to be addressed [56,119]. Secondly, the presence of some MV-associated immunogens, such as toxins and LPS can result in adverse immunological effects in humans. Selected use of strains with low toxicity [11,86] or genetic modification of the parent bacteria [61,107,109] are strategies to avoid unwanted immunogens and these factors should be considered when developing MV-based therapeutics. Furthermore, there are issues that surround the design of targeted MV drug delivery vehicles for cancer treatment. While immunogenicity of MVs is a valuable trait in vaccine development, it is an undesirable feature for a cancer therapy vehicle since an elevated immune response in a highly immunocompromised individual may be detrimental [56,119]. In addition, verification of the specificity of MV targeted drug delivery is needed to rule out toxic side effects from uptake and release into non-target tissues [124]. Collectively, overcoming these hurdles will facilitate the future design and development of innovative MV-based technologies that will lead to targeted, effective and well-tolerated therapeutics.

## 12. Summary and Conclusions

Bacterial MVs display a number of desirable qualities that can be exploited for therapeutic benefit. Harnessing the natural immunogenicity of MVs, or refining their immunogenicity through recombinant MV production, has shown great promise in novel vaccine design to treat a range of bacterial and viral diseases. The ability to display recombinant antigens on MVs by molecular techniques gives great flexibility in vaccine design. Furthermore, the potential of cell-specific targeting through display of surface receptors has the ability to revolutionize cancer treatment by enabling tumor-specific drug delivery. However, issues regarding batch consistency and purity, tissue specificity, and undesirable immunogenicity of MVs need to be resolved in order to develop safe and effective therapeutics. While research elucidating the therapeutic use and potential of bacterial MVs is still in its infancy, these early studies have paved the way for further investigation and refinement of MVs to treat a wide range of infectious, chronic and inflammatory health conditions.
